# ClusterTAD: an unsupervised machine learning approach to detecting topologically associated domains of chromosomes from Hi-C data

**DOI:** 10.1186/s12859-017-1931-2

**Published:** 2017-11-14

**Authors:** Oluwatosin Oluwadare, Jianlin Cheng

**Affiliations:** 1 Electrical Engineering and Computer Science Department, University of Missouri, Columbia, MO 65211 USA; 2 Informatics Institute, University of Missouri, Columbia, MO 65211 USA

**Keywords:** Clustering, Hi-C, Topologically associated domain (TAD), CTCF, Chromosome conformation capturing, Genome structure, Chromosome organization

## Abstract

**Background:**

With the development of chromosomal conformation capturing techniques, particularly, the Hi-C technique, the study of the spatial conformation of a genome is becoming an important topic in bioinformatics and computational biology. The Hi-C technique can generate genome-wide chromosomal interaction (contact) data, which can be used to investigate the higher-level organization of chromosomes, such as Topologically Associated Domains (TAD), i.e., locally packed chromosome regions bounded together by intra chromosomal contacts. The identification of the TADs for a genome is useful for studying gene regulation, genomic interaction, and genome function.

**Results:**

Here, we formulate the TAD identification problem as an unsupervised machine learning (clustering) problem, and develop a new TAD identification method called ClusterTAD. We introduce a novel method to represent chromosomal contacts as features to be used by the clustering algorithm. Our results show that ClusterTAD can accurately predict the TADs on a simulated Hi-C data. Our method is also largely complementary and consistent with existing methods on the real Hi-C datasets of two mouse cells. The validation with the chromatin immunoprecipitation (ChIP) sequencing (ChIP-Seq) data shows that the domain boundaries identified by ClusterTAD have a high enrichment of CTCF binding sites, promoter-related marks, and enhancer-related histone modifications.

**Conclusions:**

As ClusterTAD is based on a proven clustering approach, it opens a new avenue to apply a large array of clustering methods developed in the machine learning field to the TAD identification problem. The source code, the results, and the TADs generated for the simulated and real Hi-C datasets are available here: https://github.com/BDM-Lab/ClusterTAD.

## Background

A chromosome is known to occupy its own territory, and fold into a high-order, non-random structure in a nucleus [1]. The knowledge of the high-order organization of chromosomes is useful for the understanding of genome folding, long-range gene interactions and regulations [2], DNA replication [3], and cellular functions [4, 5]. To gain better insights into the organization of the chromosomes in a cell, a technology called the chromosome conformation capture technique such as 3C [6], 4C [7, 8], 5C [9], and Hi-C [10] has been developed to determine spatial chromosomal interaction within a chromosome region, a chromosome or an entire genome. Particularly, the Hi-C technique [10] is capable of capturing genome-wide chromosomal interactions (or contacts) by cross linking interacting DNA fragments, excising them out, sequencing them, and mapping them to a reference genome. The sequence reads obtained by the Hi-C technique are read pairs that reveal the chromosomal locations, or regions within spatial proximity to each other. By taking advantage of the high-throughput next generation sequencing techniques, the Hi-C technique can generate genome-wide, large-scale intra- and inter-chromosome contact data that can describe the spatial interactions within a genome. This genome description can be made at a detailed level, if a sufficiently deep sequencing of interacting DNA fragments is carried out. The recent study of the Hi-C data revealed that the local regions in a chromosome tend to have a lot more contacts within them than between them. These regions with more within-interaction are called Topologically Associated Domains (TAD). TADs are considered to be the structural and functional unit (or module) of a chromosome. According to [11], these TADs are unchanged irrespective of cell differentiation, and they also contain gene clusters that are co-regulated. In recent years, the detection of topological domain has become an important problem in bioinformatics, and computational biology, and as a result, several methods for TAD identification have been developed [11–17].

In this work, we formulate the TAD detection problem as grouping or clustering spatially interacting chromosomal regions into clusters. With this formulation, the TAD detection problem is tackled by unsupervised machine learning (clustering) methods. The rationale is that the chromosomal fragments within the same topological domain have many more interactions between them than those between different topological domains. Therefore, the fragments within the same topological domain tend to have similar interaction profiles than those from different topological domains. Based on this insight, we developed an algorithm to group chromosomal fragments (or regions) that have similar interaction profiles into clusters, which are used for detecting TADs. To prepare a Hi-C contact matrix data as input to a clustering algorithm, we introduce a new feature representation describing the interaction profiles of a chromosomal region, which is suitable for clustering. Our method - ClusterTAD can produce fine-scale TADs that are complementary and consistent with existing methods. Moreover, this approach opens a new avenue to apply many other well-studied clustering methods developed in the machine learning, and data mining community to the relatively new TAD detection problem.

## Methods

**Fig. 1:**
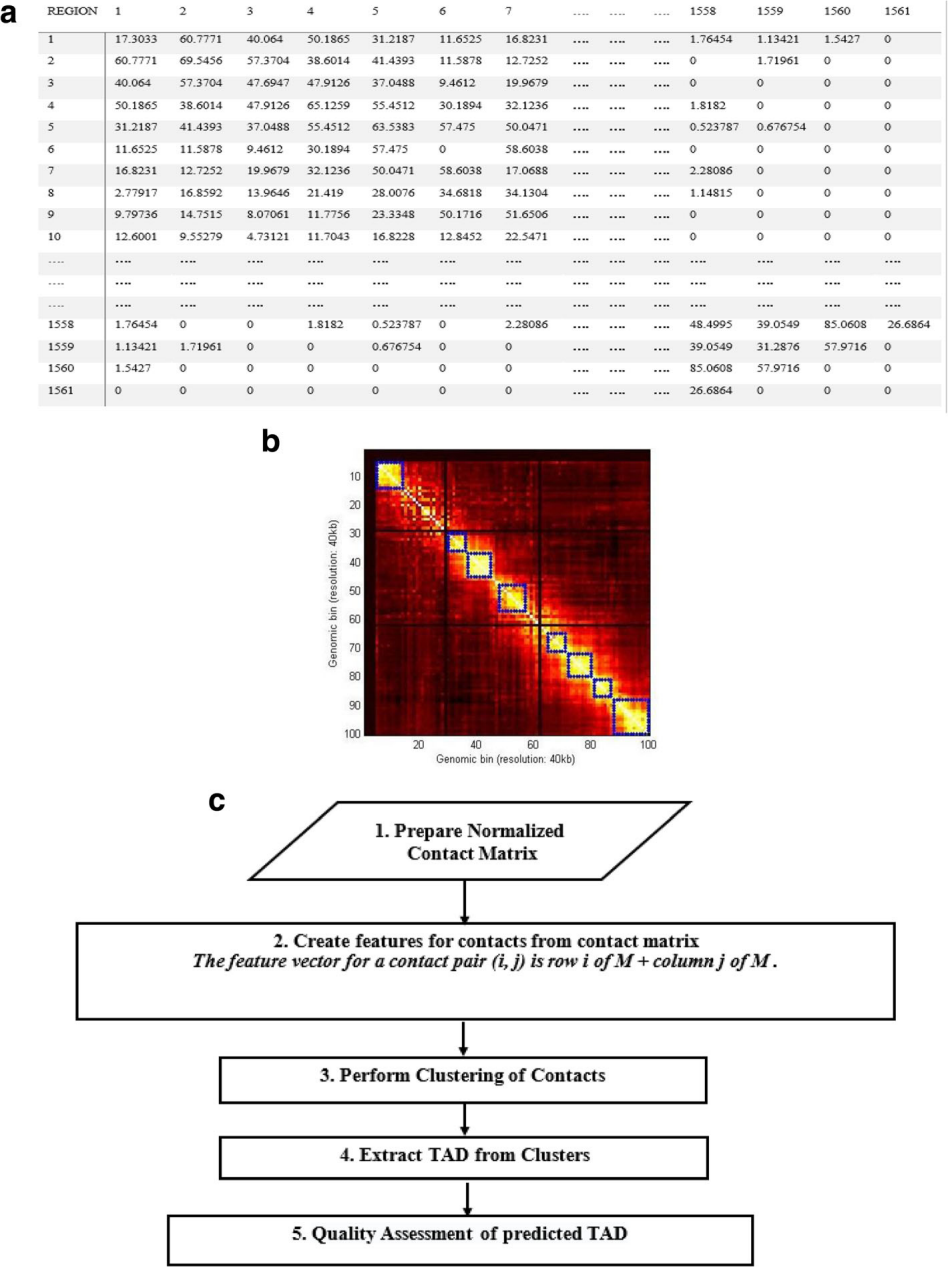
Chromosome contact matrix, TADs, and the workflow of ClusterTAD. **a** The contact matrix of Chromosome 20 of the human embryonic stem cell (hESC). The x and y-axes represent the regions of the chromosome. **b** Representation of TADs along the main diagonal of a heat map visualizing a 100 × 100 chromosomal contact matrix at 40 KB resolution. The intensity of colors represents the value of interaction frequency in the matrix. The blue squares along the main diagonal denote the identified TADs in the contact matrix. **c** The workflow of ClusterTAD

The input to our clustering-based TAD detection method (ClusterTAD) is a N by N intra-chromosomal contact matrix, M [10, 11], derived from Hi-C data, where N is the number of equal-sized regions of a chromosome. A chromosomal region is also referred to as a chromosomal bin or unit in some previous works [11, 12]. The contact matrix, M, is a square matrix that represents all the observed interactions between the regions (or bins) in a chromosome. Therefore, the value of an element in the contact matrix, represented as M[i, j], records the interaction frequency between two regions (i and j) of a chromosome. As an example, Fig. 1a shows the contact matrix of Chromosome 20 derived from the Hi-C data of the human embryonic stem cell (hESC) [18].

Generally speaking, ClusterTAD takes a Hi-C data contact matrix as input, reformats the input data, and groups the contact pairs that are spatially close to each other into the same cluster. These groups are thereafter used to identify TADs. To provide a detailed clarification of the TAD detection problem, a visual representation of the TADs in a contact matrix is shown in Fig. 1b. The squares along the main diagonal of the contact matrix are the TAD identified for this contact matrix. Figure 1c shows the workflow for ClusterTAD step by step. The specific steps of this workflow are described in detail below.

### Step 1: Prepare normalized contact matrices for chromosomes

Given a Hi-C data and a specific resolution, we generate a contact matrix for each chromosome. To reduce noise and biases, a normalization method can be used to normalize the original contact counts to create a normalized contact matrix. In this work, we used the Hi-C datasets from Dixon et al. [11], which had been binned at 40 kb resolution, and normalized for sequencing bias using the method from Yaffe and Tanay [19].

### Step 2: Create features for contacts in contact matrix

A key issue regarding clustering contacts into groups is determining the best way to define the informative features to represent each contact (i, j) involving two regions i, and j. In this work, we consider two pieces of information relevant to each contact (i,j) as its features. Firstly, all the contact data on the i^th^ row in the contact matrix, M, to represent the contact profile of region i. Secondly, all the contact data on the j^th^ column of the contact matrix, M, to represent the contact profile of region j. Therefore, the feature vector for contact M [i, j] consists of 2 N numbers, where N is the number of rows (or column) of the contact matrix. We used this feature representation because it includes all the contact profiles of the regions in contact; hence, making our feature informative and discriminative. Because a contact matrix is symmetric, only the contacts in the upper triangle of the contact matrix need to be considered. Since we only needed to group the regions along the main diagonal into clusters for TAD detection, we generated the features for only the contacts on the main diagonal to speed up clustering.

### Step 3: Clustering

Once the feature generation for the contacts along the diagonal of the contact matrix is completed, a clustering method [20–22] is needed to cluster them into groups. Different types of clustering algorithms have been developed, which can be classified into the following categories: *partitioning methods, hierarchical methods, model based methods, density-based methods*, and *grid-based methods* [23]. In this work, we applied the hierarchical clustering method, Expectation-Maximization, and K-means clustering method combined with various distance metrics on a simulated Hi-C dataset. Our results in the Result Section shows that all the methods generate comparable results. To use ClusterTAD, the number of clusters, K, is the only parameter that needs to be defined. And the presumably best K value for a dataset can be estimated automatically by ClusterTAD for user’s convenience (see the Results Section).

### Step 4: Extract TAD from contact clusters

As shown in Fig. 1b, each square (TAD) highlighted on the contact matrix contains dense contacts within them, and sparse contacts between them. Therefore, a square can be considered as the cluster of contacts that have similar contact profiles. Hence, the contact clusters identified by ClusterTAD in Step 3 can be used to identify TADs.

**Fig. 2:**
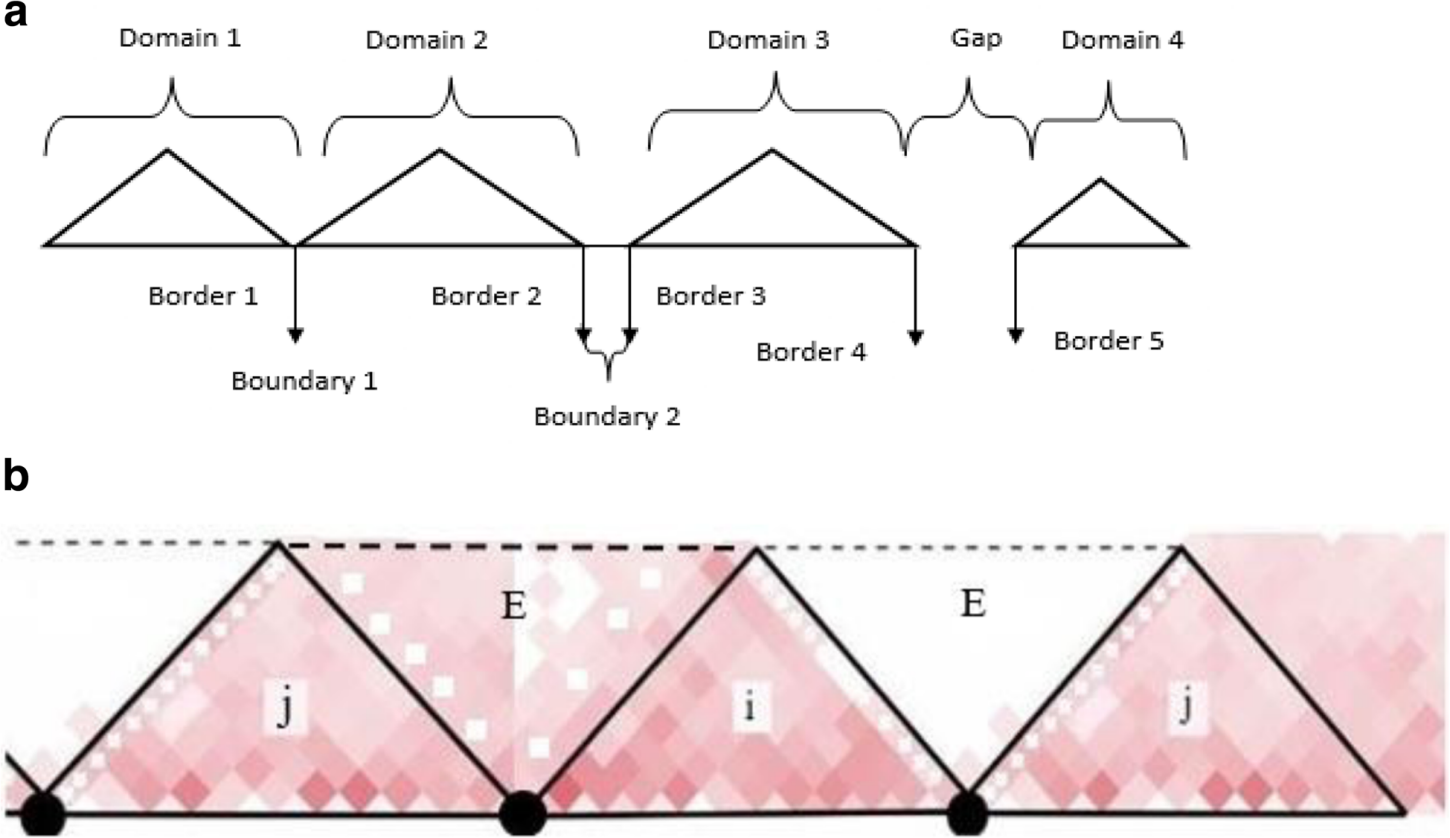
Illustration of the topologically associated domains. **a** Illustration of the basic elements related to TAD: domain, border, boundary, and gap. A domain is a TAD. A boundary is the chromosomal region between two consecutive TADs. The border marks the start/end of a domain. A gap is a point with no interaction in the contact matrix. **b** The calculation of TAD quality score. Two adjacent TADs are denoted as i and j. The area between TADs i and j that has few interactions is labeled as E. The intra(i) is the average contact frequency within a TAD (e.g. the area marked i). The inter(i, j) is the average contact frequency of the area marked as E. The difference of the two is the quality of TAD i

Once the contacts on the main diagonal are assigned into clusters, we join the consecutive contacts on the main diagonal belonging to the same cluster into segments. Based on previously reported works and experimental findings [11–14], the minimum TD size is about 180 kb. We categorized the joined segments into three groups. The segments on the main diagonal that have zero contacts are labeled as “Gap regions”. The segments greater than the minimum length are labeled as “TAD regions”. The segments that have fewer than the minimum length of a TAD are filtered out, and labelled as “Boundary regions”. Figure 2a visually explains the different types of segments defined for a dataset by ClusterTAD.

### Step 5: Evaluation of predicted TADs

An important characteristic of TADs is that, bins (regions) within a given TAD have similar contact frequency profiles, which are different from those of bins outside the TAD. Intuitively, maximizing the within-TAD similarity and minimizing the between-TAD similarity is important for evaluating the quality of TADs. Based on this property, we used the difference between the average of contact frequency of the bins in a TAD i, denoted as intra(i), and the average of contact frequency of the bins between TAD i and adjacent TAD j, denoted as inter (i, j) where |i-j| = 1 [14], to assess the quality of TAD assignments. This TAD quality score is represented in Eq. 1 and visually represented in Fig. 2b.

1 $$ \mathrm{TAD}i\ \mathrm{Quality}=\kern0.5em intra(i)- inter\left(i,j\right) $$

Equation 1 is used to compute the quality of each TAD defined for a dataset. The overall quality score for a set of TADs defined for a contact matrix is their average quality score. Consequently, the set of TADs with the highest quality score is chosen as the representative domain set for a chromosome.

### Datasets

The simulated dataset from Wang et al., 2015 [13] is a 30-bin Hi-C contact matrix, in which the contacts were simulated from a chromosome structure with predefined topological domains. The contact matrix and the predefined domains of the simulated dataset were downloaded from [13].

The real Hi-C dataset used in this study is the Hi-C data of two mouse cells: the mouse embryonic stem cell and the cortex cell at a bin resolution of 40 kb. The normalized contact matrices for these cells are available at [18].

The ChipSeq data used to analyze the enrichment of CTCF and other histone modifications is from Shen et al. (32). The raw data is available in the Gene Expression Omnibus (GEO) database with the GEO accession ID GSE29184. The extracted peaks for this ChipSeq data can be downloaded from [24].

## Results and discussion

### Determination of the parameter of ClusterTAD

ClusterTAD needs a single parameter, K (the number of clusters), to compute the set of TADs for a chromosome contact matrix. For most clustering algorithms, it is always important to find the “best” K parameter for a particular dataset, because this parameter influences the quality of the cluster analysis. However, it is worth mentioning that the definition of the “best” K parameter is usually subjective because the “right” number is often ambiguous [23]. Here, we use two well-known approaches to estimate the “best” possible value of K parameter as follows.

1. A method proposed by Han et al. [23] assumes that each cluster for a dataset has about $\sqrt{2n}$ points for a dataset of n points, and the number of clusters can be estimated using Eq. (2).

2 $$ \mathrm{K}=\sqrt{\frac{n}{2}} $$

To allow some flexibility, we created a window around this estimated K value. We set the lower limit of the estimated number of clusters equal to K – 10, and upper limit equal to K + 10. We used this method as the default one for ClusterTAD for the real Hi-C data.

2) The elbow method [25, 26] is one of the oldest methods to determine the number of clusters. It chooses the number of clusters, K, such that increasing the number of clusters (K + 1, K + 2, …) results in no significant change in the within-cluster variance. Usually, it starts at K = 2 and increases K with an increment of 1 to an upper limit, which is usually the number of instances in the dataset. The elbow is regarded as the point where adding another cluster does not improve the quality of clustering much. The elbow method can be computationally costly for large datasets, but extremely useful and efficient for small datasets.

### Evaluation of the clustering quality

We used two different statistical evaluation measures to assess the quality of the clusters of chromosomal contacts.

1. **The Davies-Bouldin index** [27] (DBI). DBI is defined as.

$$ \mathrm{DBI}=\frac{1}{N}\ {\sum}_{i=1}^N{D}_i $$

where $$ {D}_i={\mathit{\max}}_{j\ne i}{R}_{i,j},{R}_{i,j}=\frac{d_i+{d}_j}{d_{i,j}} $$

Where d_i_ is the distance of elements in cluster i to its centroid. d_i,*j*_ is the measure of the separation of clusters i, and j, equal to the distance between the centers of clusters i and j. A lower DBI score is preferred.

(2). **The Silhouette Index** [28] (SI). SI is defined as.

$$ \mathrm{SI}=\frac{1}{N}{\sum}_{i=1}^N\frac{1}{\left|{c}_i\right|}{\sum}_{j\in {c}_i}{s}_j $$

where $$ Sj=\frac{b_j-{a}_j}{\mathit{\max}\left\{{a}_j,{b}_j\right\}} $$

Where a_j_ is the average distance of data point j to all other data points within the same cluster (C_i_). A smaller a_j_ value implies a better cluster assignment. b_j_ is the average distance of data point j to the data in the next best fit cluster for it or to another cluster with lowest average distance to j. The Silhouette coefficient value ranges between −1 and 1. A higher SI score is considered better.

### Assessment on the simulated dataset

**Fig. 3:**
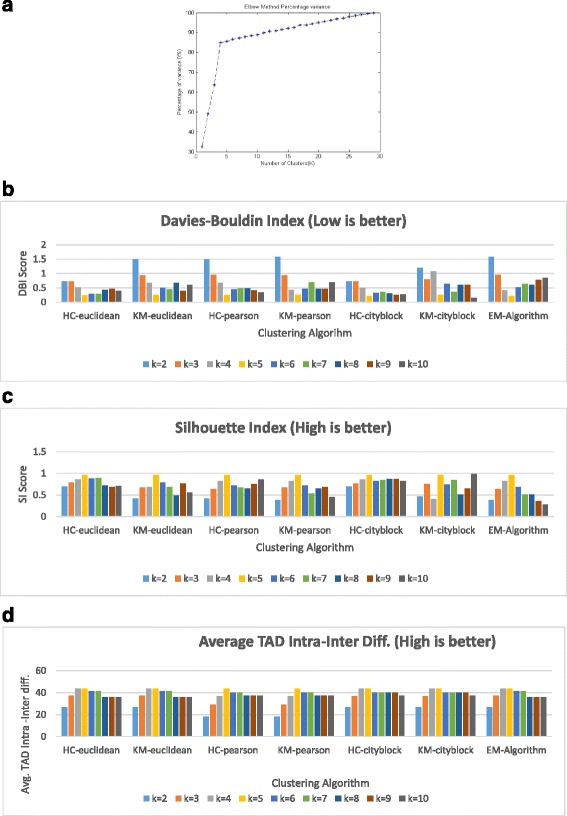
The results on the simulated dataset. **a** An elbow plot for the clustering results of ClusterTAD on the simulated dataset. The percentage of within-cluster variance is plotted against the number of clusters. The elbow point is at K = 5. **b** The Davies-Bouldin index (DBI) for the different clustering algorithms. **c** The Silhouette Index (SI) for the different clustering algorithms. **d** The average Intra-Inter difference scores for the TADs extracted by ClusterTAD with different combinations of clustering algorithms and distance metrics: HC-eulcidean, KM-eulidean, HC-pearson, KM-pearson, HC-cityblock, KM-cityblock, and the EM. HC denotes the hierarchical clustering algorithm, KM the K-means algorithm, and EM the expectation maximization algorithm. HC-euclidean represents the combination of the hierarchical clustering algorithm with Euclidean distance metric

We first evaluated our method on a simulated Hi-C contact matrix dataset [13]. We applied ClusterTAD on this dataset and compare its results with the known true results. We used three clustering algorithms with ClusterTAD to the dataset, including the k-means (KM) method, the hierarchical clustering (HC), and the Expectation Maximization (EM) algorithm. For the KM, and HC algorithms, we applied three distance metrics: the Euclidean-distance, the Pearson correlation distance, and the city-block distance. These algorithms require the number of cluster to be specified for them to be used. Firstly, using the Han et al. method, the number of clusters, K, can be estimated from the number of data points (n) in the dataset. Using Eq. (2), we estimated the initial number of Cluster (K) to be 4. A window around the estimated K value specifies the range of the potential numbers of clusters to be tested in our clustering analysis. Secondly, using the elbow method, we plot the percentage of variance against the number of clusters for the dataset (Fig. 3a). From the plot, we can infer that the elbow point is at 5.

Once the number of cluster is defined, we performed the clustering on the simulated dataset using the three clustering algorithms above. We evaluated the quality of the clustering results using the Davies-Bouldin index (DBI) and Silhouette Index (SI). The results are shown in Fig. 3b, c. The best clustering quality is achieved at K = 5 for both DBI (Fig. 3b and SI (Fig. 3c) measures for most combinations of the algorithms and distance metrics.

Once the clustering was done, we applied ClusterTAD to extract the TADs from the clustering results of all the algorithms, respectively. As described earlier, once the TAD is extracted, Eq. (1) is used to evaluate the quality of the TADs. Figure 3d, shows the Intra-Inter difference quality scores of TADs. The highest intra-inter difference was achieved with the different clustering algorithms at K = 5 regardless distance metrics used, showing the quality of TADs is consistent with that of the clustering results.

**Fig. 4:**
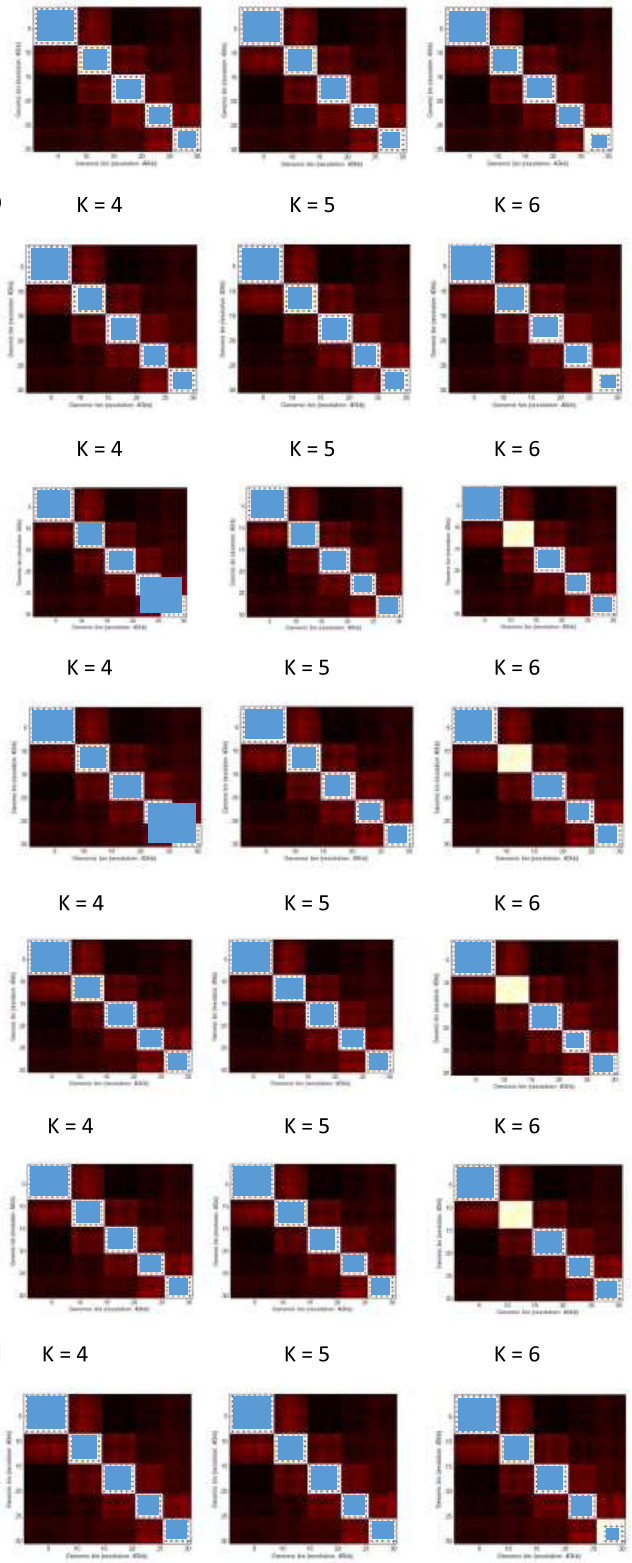
– The visualization of the TADs extracted for one chromosome contact map in the simulated dataset. Rows a to g represents the TADs extracted for K = 4, K = 5 and K = 6 (from left, middle to right) for the following combinations of clustering algorithms and distance metrics: (**a**) HC-eulcidean, (**b**) KM- eulidean, (**c**) HC-pearson, (**d**) KM-pearson, (**e**) HC-cityblock, (**f**) KM-cityblock, and (**g**) EM. HC denotes the hierarchical clustering algorithm, KM the K-means algorithm, and EM the expectation maximization algorithm. HC-euclidean denotes the combination of the hierarchical clustering algorithm with the Euclidean distance metric. The left column visualizes the TADs extracted by the seven algorithms when K = 4, the middle columns the TADs extracted when K = 5, and the right column the TADs extracted when K = 6. A TAD region identified on each contact heatmap is denoted by a blue square within the blue dots along its diagonal. The blue dots represent the boundary of a TAD region. The white squares along the diagonals are unrecognized TADs

Figure 4a-g visualizes the TADs identified at K = 4 (left), K = 5 (middle) and K = 6 (right) by HC-euclidean, KM-eulidean, HC-pearson, KM-pearson, the HC-cityblock, KM-cityblock, and EM algorithm, respectively. The TADs are represented as blue squares on the contact heat maps. A TAD identified on each of the contact matrix is the blue region within the blue dots along the diagonal of the contact matrix heat map. These dots represent the boundary of the TAD, which forms squares on each of the contact matrix. Within this boundary are regions with more interactions to each other than to other areas on a contact matrix. Table 1 lists the TADs identified by each of the seven different algorithms visualized in the Fig. 4. With this visualization, we were able to observe the consistency between the quality scores of TADs in Fig. 3, and the true accuracy of TADs shown in Fig. 4. The quality score is higher when the TAD result is more accurate. For instance, HC-euclidean at K = 4 and 5 in Fig. 3d have the highest quality score, and their corresponding TADs are the same as the true TADs (Fig. 4a left and middle). It is observed from Fig. 4 that the seven different algorithms identify the same set of TADs when the number of clusters (K) equals to 5, which is consistent with the results in Fig. 3 where the TADs produced by the seven algorithms have similar quality scores when K equals to 5.

**Table 1:** The lists of TADs identified by the seven different algorithms in Fig. 4

Algorithm	K = 4	K = 5	K = 6
a	{(1,8), (9,14), (15,20), (21,25), and (26,30)}.	{(1,8), (9,14), (15,20), (21,25), and (26,30)}.	{(1,8), (9,14), (15,20), (21,25), and (27,30)}.
b	{(1,8), (9,14), (15,20), (21,25), and (26,30)}.	{(1,8), (9,14), (15,20), (21,25), and (26,30)}.	{(1,8), (9,14), (15,20), (21,25), and (27,30)}.
c	{(1,8), (9,14), (15,20), and (21,30)}.	{(1,8), (9,14), (15,20), (21,25), and (26,30)}.	{(1,8), (15,20), (21,25), and (26,30)}.
d	{(1,8), (9,14), (15,20), and (21,30)}.	{(1,8), (9,14), (15,20), (21,25), and (26,30)}.	{(1,8), (15,20), (21,25), and (26,30)}.
e	{{(1,8), (9,14), (15,20), (21,25), and (26,30)}.	{(1,8), (9,14), (15,20), (21,25), and (26,30)}.	{(1,8), (15,20), (21,25), and (26,30)}.
f	{(1,8), (9,14), (15,20), (21,25), and (26,30)}.	{(1,8), (9,14), (15,20), (21,25), and (26,30)}.	{(1,8), (15,20), (21,25), and (26,30)}.
g	{(1,8), (9,14), (15,20), (21,25), and (26,30)}.	{(1,8), (9,14), (15,20), (21,25), and (26,30)}.	{(1,8), (9,14), (15,20), (21,25), and (27,30)}.

The table contains the lists of TADs extracted for K = 4, K = 5 and K = 6 (from left, middle to right) by the seven algorithms: (a) HC-eulcidean, (b) KM-eulidean, (c) HC-pearson, (d) KM-pearson, (e) HC-cityblock, (f) KM-cityblock, and (g) EM. HC denotes the hierarchical clustering algorithm, KM the K-means algorithm, and EM the expectation maximization algorithm. HC-euclidean denotes the combination of the hierarchical clustering algorithm and the Euclidean distance metric. A TAD is represented as {start, end}, where “start” is the TAD start region, and “end” is the TAD end region. The best TAD set for the synthetic data is {(1, 8), (9, 14), (15, 20), (21, 25), and (26, 30)}

### Assessment of ClusterTAD on real hi-C datasets

**Fig. 5:**
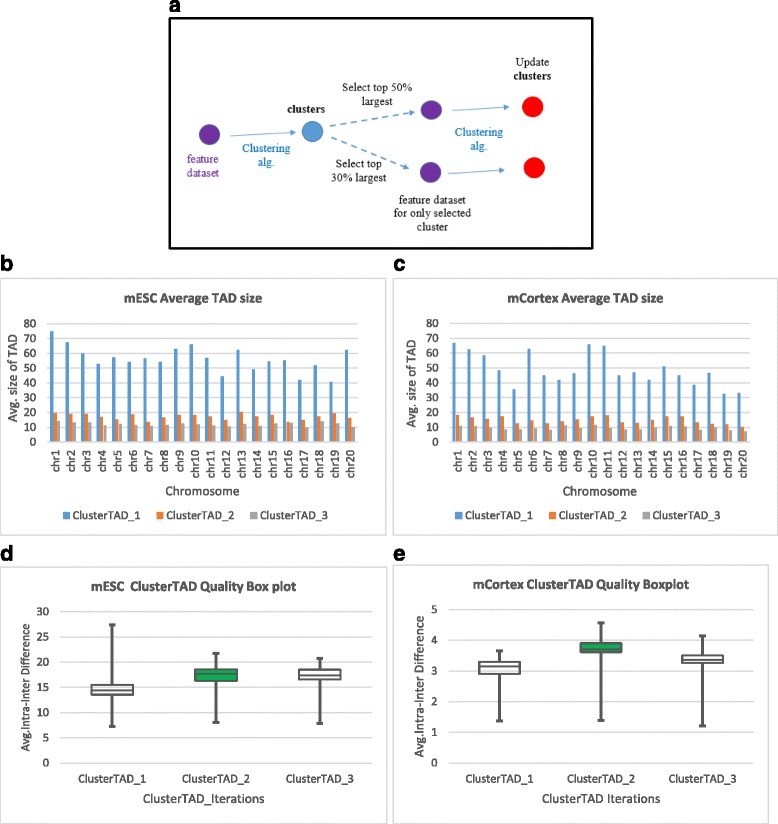
Evaluation on a real Hi-C dataset. **a** The workflow of the iterative application of ClusterTAD. **b** The average size of TADs identified for the mouse embryonic stem cell by three rounds of clustering of ClusterTAD (ClusterTAD_1, ClusterTAD_2, and ClusterTAD_3). **c** The average size of TADs identified for the mouse cortex cell by three rounds of clustering of ClusterTAD. **d** The box plot of the quality scores of TADs extracted for the mouse embryonic stem cell by the three rounds of clustering of ClusterTAD. **e** The box plot of the quality scores of TADs extracted for the mouse Cortex cell for the different clustering operations performed by ClusterTAD

We tested ClusterTAD on the Hi-C data of two mouse cells: the mouse embryonic stem cell and the mouse cortex cell at a bin resolution of 40 kb. We used the K-means algorithm with Euclidean distance metric for the clustering performed on the real Hi-C datasets. The first round of the application of ClusterTAD resulted in large, coarse clusters, and consequently large TADs. As illustrated in [11–14] that large TADs often have lower average interactions within TADs, in order to improve cohesiveness of TADs, we applied another round of clustering to large clusters generated in the first round. Figure 5a shows the workflow of multiple steps of clustering with ClusterTAD. Re-clustering of the existing clusters generates sub-clusters. To identify the set of clusters to be re-clustered from the results of the first round of clustering (ClusterTAD_1), we ranked the clusters generated from ClusterTAD_1 based on the number of points (regions) in each cluster. Then we selected the top 30% or 50% largest clusters for re-clustering with the same algorithm of ClusterTAD, such that at least 50% of clusters in the current round will be kept. The second round of clustering is denoted as ClusterTAD_2. The third and also last round of clustering operation is called ClusterTAD_3.

Figure 5b, c shows the average size of TADs generated in the three rounds of clustering. The average size of TADs decreases from one round to next round as expected. Figure 5d, e reports the inter-intra interaction frequency scores of TADs of the three rounds. ClusterTAD_2 consistently achieved the highest average score. Though ClusterTAD_3 has smaller TADs than ClusterTAD_2, its quality score is lower than ClusterTAD_2.

**Fig. 6:**
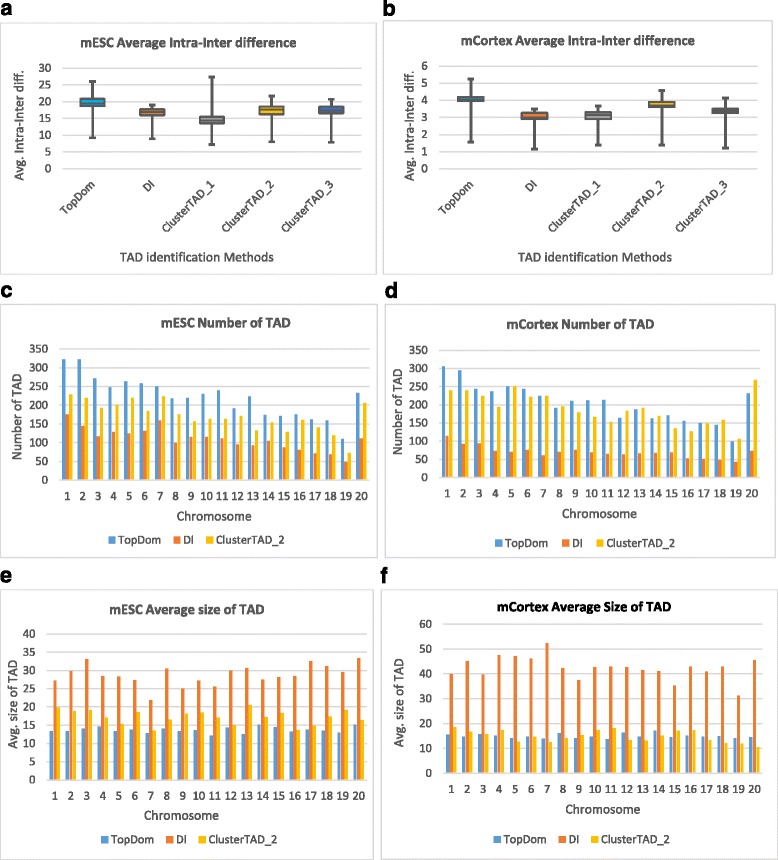
Comparison of the quality scores, numbers and average sizes of TADs identified by TopDom, DI, and ClusterTAD on two mouse cell lines. **a, b** The comparison of the intra-inter difference scores; (**c, d**): the number of TADs, and (**e, f**) the average size of TADs for the mESC and mCortex cells respectively

We compared ClusterTAD with the two other widely used methods: the directionality index (DI) method [11] and the TopDom [14] methods on the mouse Hi-C datasets. The results of DI and TopDom were obtained from their published data. Figure 6 shows the quality scores of TADs, the number of TADs, and the average size of TADs of the three methods. Generally speaking, DI detects TADs of larger sizes, TopDom identifies TADs of smaller size, and ClusterTAD produces the results in the middle. Figure 6e, f shows the average size of TADs identified by TopDom, DI, and ClusterTAD for the mESC, and mCortex cells respectively. The average size of the TADs produced by ClusterTAD is significantly smaller than DI, but somewhat larger than TopDom (Fig. 6e) or comparable to it (Fig. 6f). This is consistent with the observation that DI tends to detect TAD with large sizes, while TopDom tends to identify smaller TADs called sub-TADs. Since ClusterTAD tends to break larger TADs into smaller TADs to improve their cohesiveness, the average size of TADs identified by ClusterTAD is between DI and TopDom, while leaning more toward TopDom. Since the TADs identified by ClusterTAD and TopDom have a smaller size, they tend to have higher inter-intra interaction frequency scores.

**Fig. 7:**
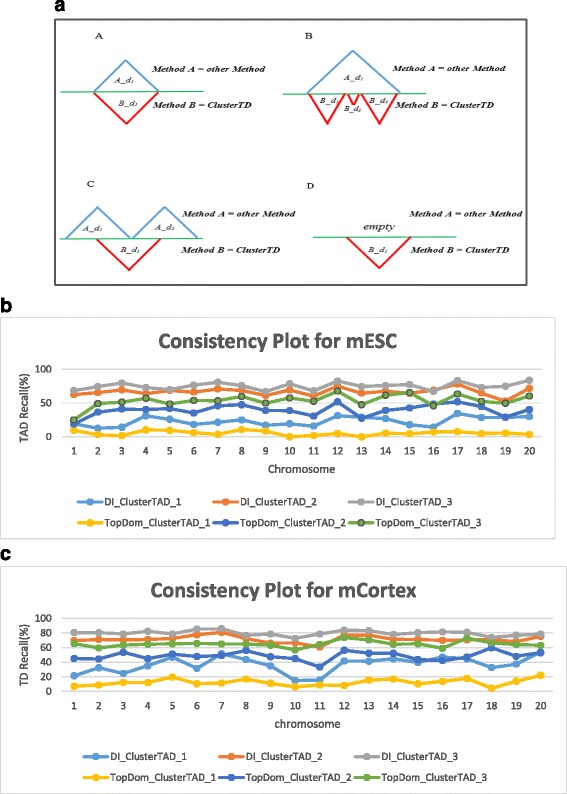
The analysis of the consistency between TADs identified by ClusterTAD and other methods on the two mouse cell lines. **a** Four different cases in which TADs detected by two different methods are compared with each other. Case A: This refers to the case in which the TAD identified in method B exactly matches those from another method A. The TADs detected by the two methods have the same boundaries. Case B: This refers to the case in which a TAD detected by method A contains two or more domains detected by method B. The smaller TADs detected by method B are called sub-TAD of the TAD detected by method A. Case C: This represents the conflicting case in which the domain detected by method A does not match or contain the domains detected by method B even though there is some overlap between them. Case D: This refers to the rare case in which the region is not assigned to a TAD by method A, but is assigned by a TAD by method B. **b** The percentage of TADs detected by ClusterTAD for the mESC cell line that were also detected by TopDom and DI. (**c**) The percentage of TADs detected by ClusterTAD for the mCortex cell line that were also detected by TopDom and DI

We assessed how consistent the TADs detected by ClusterTAD are with those by DI and TopDom. The consistency check was carried out according to the method described in Fig. 7a. A TAD detected by method A is considered also detected by method B if the similarity between the TADs by method A and the TADs by method B falls in Case A or Case B in Fig. 7a, b, c shows the percentage of TADs detected by ClusterTAD that were also detected by the other methods. A higher percentage of TADs identified by ClusterTAD was found by DI than by TopDom probably because the TADs predicted by TopDom were generally smaller. Overall, the three methods appear to produce the complementary results on the dataset.

### Validation of ClusterTAD by the enrichment analysis of CTCF binding sites and histone modification marks in domain boundaries

Topologically Associated Domains (TADs) are known to have a high level of interactions within them, compared to those between them. Each domain is separated from each other by domain boundaries. Therefore, TAD boundaries can be regarded as an insulator that restricts interaction between a TAD and its adjacent TADs [11, 29]. And TAD boundaries are also known to have an enrichment of binding sites of CTCF – a genome architectural protein [15–17, 29–33]. The binding sites of CTCF can be determined by a chromatin immunoprecipitation (ChIP) sequencing (ChIP-Seq) technique. We validated the result obtained from ClusterTAD by checking the enrichment of CTCF at the boundary between TADs for each of the mouse cells.

**Fig. 8:**
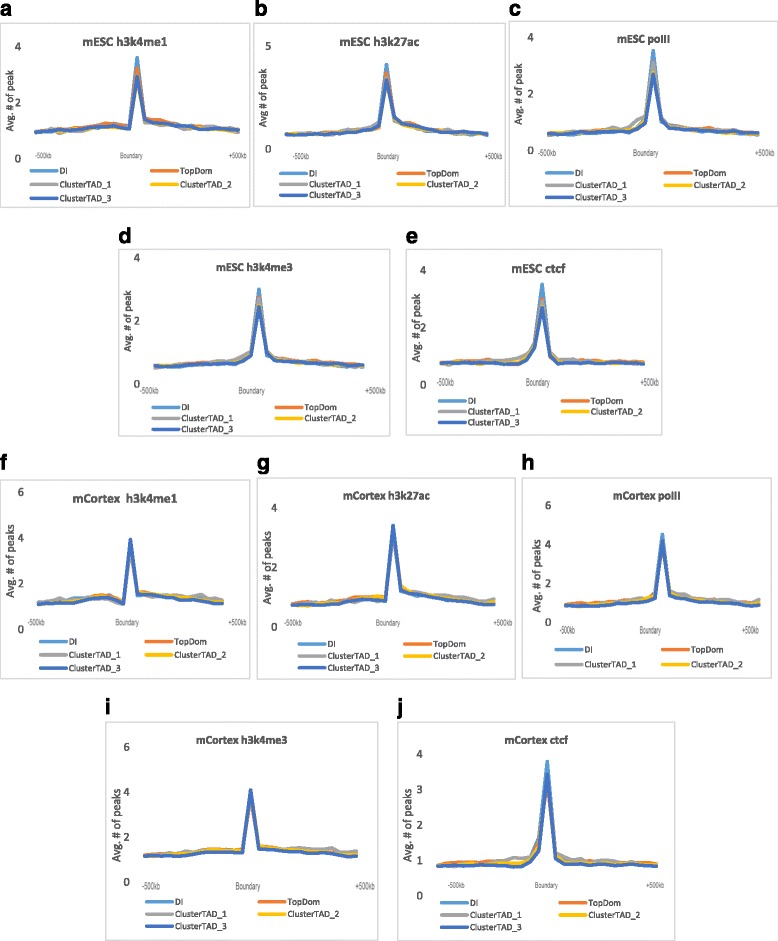
– The enrichment analysis of active histone modification marks and CTCF binding sites at the domain boundary. The average peak number of active histone modification marks (promoter marks (Polymerase II and H3K4me3) and enhancer marks (H3K4me1 and H3K27ac) and CTCF binding sites at the boundary regions identified by TopDom, DI and ClusterTAD for mouse Embryonic Stem Cell line (mESC) (**a-e**) and the mouse cortex cell line (mCortex) (**f-j**)

We used the dataset of the predicted cis-regulatory elements extracted from Chip-Seq data by Shen et al. [34] to assess the abundance of CTCF binding sites at the domain boundaries of TADs. Though CTCF binding sites are largely found at domain boundaries, CTCF are also associated with some active histone modification to form the insulation in the domain boundaries. Hence, in addition to studying the CTCF enrichment in the boundaries, we also investigated the enrichment of promoter marks: RNA Polymerase II and H3K4me3, and enhancer-marks (H3K4me1 and H3K27ac). Using the Chip-Seq data, the peaks for the CTCF and histone modification marks were identified using MACS [35] with the default parameters and filtered by a *p*-value of 0.00001. Figure 8 shows the occurrence of high number of peaks (enrichment) for CTCF binding sites, and the histone modification marks at the boundaries of TADs identified for the two mouse cells by ClusterTAD, DI and TopDom, validating that the domain boundaries recognized by ClusterTAD are biologically relevant. According to the enrichment analysis in Fig. 8, there was a reduction in the average number of peaks for the enhancer mark H3K27ac in the mouse cortex cells than in the mESC cells, which is consistent with the previous discovery in [14]. In addition, the H3K4me1 peak enrichment in the mCortex cells was slightly higher than in the mESC cells. The enrichment of CTCF, H3K27ac, and H3K4me1 in the predicted TAD boundaries suggests that they may act as an insulator to separate TADs [11, 29]. The previous studies show that enhancers could activate transcription by bringing accessory transcription-related factors to gene promoters within their spatial proximity [36], even though the promoters may be sequentially far away from the enhancers in the linear genome sequence [37]. Hence, the high enrichment of the enhancer and promoter marks in the boundary regions suggests that some TAD boundary regions can be transcription activation sites.

## Conclusions

We introduce ClusterTAD, a new clustering based method, to detect TADs from Hi-C data. ClusterTAD employs standard clustering algorithms to extract topological domains from Hi-C contact data. We show that ClusterTAD is consistent and complementary with existing methods. The TAD boundaries identified by ClusterTAD are validated by the enrichment analysis of CTCF binding sites and histone modification marks. It is easy to use ClusterTAD since it only requires one parameter – the number of cluster, and the parameter can be estimated automatically from the data. Moreover, ClusterTAD can be iteratively applied to divide larger clusters into small ones, which can be used to identify both large TADs and smaller sub-TADs. Finally, by formulating the TAD detection problem as a classic clustering problem through a novel representation of chromosomal contacts, an array of clustering methods in the field of machine learning can be applied to address the problem. We expect more sophisticated clustering algorithms will be used to improve TAD detection in the future.

## Abbreviations

ChIP: Chromatin immunoprecipitation
ChIP-Seq: Chromatin immunoprecipitation sequencing
CTCF: 11-zinc finger protein or CCCTC-binding factor
IF: Interaction frequency
TAD: Topologically associated domain
